# Prognostic factors for tumor recurrence in patients with clinical stage I seminoma undergoing surveillance—protocol for a systematic review

**DOI:** 10.1186/s13643-015-0167-3

**Published:** 2015-12-18

**Authors:** Frank Kunath, Annabel Spek, Katrin Jensen, Friedemann Zengerling, Stefanie Schmidt

**Affiliations:** Department of Urology, University Hospital of Erlangen, Erlangen, Germany; Department of Urology, University Hospital of Munich, Munich, Germany; Institute of Medical Biometry and Informatics, University of Heidelberg, Heidelberg, Germany; Department of Urology, University Hospital of Ulm, Ulm, Germany; UroEvidence@Deutsche Gesellschaft für Urologie, Berlin, Germany

**Keywords:** Testicular cancer, Seminoma, Relapse, Rete testis invasion, Tumor diameter, Adjuvant therapy, Surveillance, Systematic review, Prognostic factor

## Abstract

**Background:**

Testicular cancer is primarily treated with the surgical removal of the affected testis. About 50 % of testicular cancer patients present with a stage I seminoma. If no chemo- or radiotherapy as adjuvant treatment is initiated after orchiectomy, 15–20 % of these patients will develop metastases. Although adjuvant treatment is effective in reducing the relapse risk, there is rising concern about overtreatment of these patients. Prognostic factors at primary diagnosis might have the potential to identify patients at higher risk of tumor relapse, allowing to guide individual therapy and to avoid overtreatment. Therefore, we aim to synthesize the available evidence on tumor or patient characteristics as possible prognostic factors for cancer recurrence in patients with clinical stage I seminoma.

**Methods/design:**

We will conduct a broad systematic review to analyze what prognostic factors predict cancer recurrence in patients with a first time diagnosis of clinical stage I seminoma, who received no adjuvant chemo- or radiotherapy after orchiectomy. The literature search will comprise MEDLINE, Web of Science, the Cochrane Central Register of Controlled Trials (CENTRAL), and the conference proceedings of the American Society of Clinical Oncology (ASCO), American Urologic Association (AUA), and European Urologic Association (EAU) Annual Meetings. Prospective and retrospective longitudinal studies reporting on prognostic factors for cancer recurrence will be considered. We will consider the wealth of any candidate clinical or pathological prognostic factor reported in the literature. Our outcome of interest will be tumor recurrence at a minimum of 2 years follow-up. Study screening, data extraction, and quality assessment will be done by two reviewers independently. Hazard ratios will be used to measure the relationship between the potential prognostic factor and tumor recurrence. Meta-analyses will be conducted with sufficiently homogeneous studies and separately with respect to study design, by using the random-effects generic inverse variance model.

**Discussion:**

Limitations and strengths will be discussed in our review, and the results will be put into context with other studies in this field. Our results will help to guide evidence-based decision-making on patients with clinical stage I seminoma, allowing a better adjustment of therapies with regard to the individual patient’s risk. Our findings will furthermore help to formulate recommendations for future research.

**Systematic review registration:**

PROSPERO CRD42014009434

## Background

Testicular cancer is primarily treated with inguinal orchiectomy, that means the surgical removal of the affected testis. Pathological examination reveals histology of a seminoma in about 60 % of the cases, with an increasing trend [1]. About 80 % of the diagnosed seminomas are classified as clinical stage I. This stage is characterized by the absence of metastases on radiologic examination and by normal or normalized testis tumor markers after orchiectomy. Data from large case series show that despite normal radiologic and laboratory examinations on primary diagnosis, about 15–20 % of stage I seminoma patients will develop a tumor relapse in their further live [2]. Therefore, early additional (adjuvant) therapies, namely adjuvant chemotherapy and adjuvant irradiation of the posterior abdominal wall, are used for stage I patients because they are able to lower the relapse risk to percentages <5 % [3, 4]. Another option for patients with stage I seminoma is a surveillance strategy, which consists of a close follow-up of affected patients rather than administering any active medical intervention. Hence, surveillance, adjuvant radiation and adjuvant chemotherapy are the three therapeutic options for patients with clinical stage I seminoma.

Recent data suggest that radio- and chemotherapy harbor relevant long-term side effects, which often have been neglected in the past [5, 6]. These long-term data on chemo- or radiotherapy imply a more distinguished point of view on adjuvant therapy regimens. The ideal approach for stage I seminoma patients is to minimize overtreatment without exposing the patient to an unnecessary high risk of tumor relapse. In reality, this is difficult to achieve, but prognostic factors at primary diagnosis might have the potential to identify patients at higher risk of tumor relapse. In clinical practice, they can help to guide individual treatment and lead to reduced rates of overtreatment.

In 2002, Warde et al. published the results of a retrospective study investigating the prognostic value of histopathological findings in the primary tumor specimen for relapse. They found a tumor diameter greater than 4 cm and infiltration of the rete testis being significantly associated with tumor relapse [7]. The same working group was unable to validate these two risk factors in a prospective study [8]. Ensuing prospective studies led to conflicting results regarding the value of tumor diameter and rete testis infiltration [9, 10]. Both candidate prognostic factors have been used by some physicians in clinical decision making for or against adjuvant therapy, especially in favor of surveillance if both risk factors are absent. In contrast to patients with stage I non-seminoma -where pT2 stage is an accepted risk factor-, the guidelines of the European Association of Urology (EAU) and the European Society of Medical Oncology (ESMO) do not clearly recommend these prognostic factors for the choice of treatment for stage I seminoma patients [11, 12]. There were further efforts -sometimes only in retrospective series or on a small number of patients- to identify other candidate prognostic factors that are better suited to facilitate therapy guidance. A number of molecular examinations on primary tumor tissue or patient peripheral blood for different markers have been done, but none of them has entered the routine of clinical decision-making or even the therapy guidelines.

### Why it is important to do this review

The continuous discussion on different candidate prognostic factors, of which some -despite lacking evidence- have partly entered in clinical practice, leads to relevant doubts and uncertainties in seminoma patients and the doctors engaged in their treatment. Therefore, it seems important to systematically review, evaluate and summarize the existing information on prognostic factors in stage I seminoma to guide clinical decision making on further treatment strategies and to inform and facilitate future intervention research.

## Method/design

### Objectives

To synthesize the available evidence on the association between primary tumor as well as clinical patient characteristics as prognostic factors and cancer recurrence in patients with clinical stage I seminoma, who received no prior adjuvant therapy.

### Review design

We will conduct a broad systematic review (as opposed to a focused review that investigates evidence on only one prognostic factor) to analyze what are the candidate factors that predict cancer recurrence in clinical stage I seminoma patients.

The conduction of systematic reviews of prognostic factors is challenged by the selective reporting of prognostic factor studies [13], eventually leading to false-positive results, as well as by variations in methodology and reporting of findings among primary studies [14]. To deal with these challenges, we will follow the work done by the Prognostic Research Strategy (PROGRESS) group that developed methods for prognosis research studies [15]. Furthermore, we will follow the reporting guidelines of Preferred Reporting Items for Systematic Reviews and Meta-analyses (PRISMA) for systematic reviews [16] and Reporting Recommendations for Tumor Marker Prognostic Studies (REMARK) for (tumor marker) prognostic studies [17].

### Types of studies

We will include prospective and retrospective longitudinal observational studies investigating the prognosis of patients with clinical stage I seminoma. We will also include intervention studies, if they include a surveillance group and if results are reported separately for this group. Studies are eligible for inclusion if they report on patients diagnosed with stage I tumor and provide data with a minimum follow-up (median or mean) of 2 years. A minimum follow-up of 2 years is required to exclude relevant uncertainties of possible diagnostic factors, as about 30 % of seminoma relapses are diagnosed in the second year of follow-up and a 2-year follow-up covers at least about 75 % of all relapses [2]. Exploratory studies as well as confirmatory studies will be considered [18], if they provide data on the association of clinical or pathohistological factors with tumor recurrence. Exploratory and confirmatory studies will be treated differently in the analyses as they provide different levels of evidence. We will also consider studies published as abstracts; their limited significance will be considered in the quality assessment process. Study inclusion will not be restricted by publication status. We will exclude reviews, case reports, editorials and comments.

### Types of participants

We will include patients with clinical stage I seminoma, defined as the absence of metastasis shown with radiological imaging and normal or normalized values for the tumor marker Beta-human chorionic gonadotropin (betaHCG). Eligible are patients of all ages, ethnicity and comorbidity with a first time diagnosis of seminoma stage I, who received no prior adjuvant treatment after orchiectomy. We will exclude patients who received any adjuvant therapy (e.g., chemo- or radiotherapy).

### Types of prognostic factors

We will not focus at a specific prognostic factor but will consider the wealth of any candidate clinical or pathological prognostic factor reported in the literature. So far, especially tumor size (>4 cm) and tumor infiltration into rete testis have been reported to have a possible prognostic value in predicting tumor recurrence in seminoma patients at clinical stage I [7]. If we identify any other candidate prognostic factor during our review process, we will also include it in our analyses.

### Outcome measures

Our outcome of interest will be tumor recurrence during the observed follow-up period, as the risk for tumor recurrence is the most important factor for decisions on adjuvant therapy regimens. Tumor recurrence will be assessed as reported by the authors, regardless of the mode of detection (radiologic finding, histologic specimen, tumor marker elevation) at the described time points.

### Search methods for identification of studies

We will conduct a systematic review of the literature in different electronic biomedical databases. Our search will be complemented by additional handsearching of meeting communications of the major conferences of this topic. The searches were conducted in January 2015. A final update search will be done before submitting the final review draft for publication.

#### Electronic searches

Specific search strategies will be conducted for each of the following databases: MEDLINE (from 1946 onwards), Web of Science (from 1946 onwards), and the Cochrane Central Register of Controlled Trials (CENTRAL) (from 1995 onwards). The search strategies are shown in Appendix 1, Appendix 2, and Appendix 3. Search terms will combine keywords and free text. No date or language restrictions will be applied.

#### Searching other resources

We will handsearch the conference proceedings of American Society of Clinical Oncology (ASCO), the Annual Meeting of the European Association of Urology (EAU), and the American Urologic Association (AUA) (American Urologic Association) from 2008 onwards. Additionally, we will review the reference lists of the full texts of potential relevant studies.

### Data collection and analyses

#### Selection of studies

Articles will be selected for inclusion by title and abstract review, performed by two authors independently. The full text of the potentially relevant references will be reviewed by the same authors independently, too. We will provide a PRISMA study flow diagram with reasons for exclusion at full text screen level. Discrepancies will be resolved by discussion or by consulting a third review author.

### Data extraction and management

Data extraction will be done independently by two review authors. Disagreement will be resolved through discussion or by consulting a third review author. The data extraction form is inspired by the work of Moons et al. and adapted for our purpose [19]. The following data will be extracted: source of data (study design, study dates, sample size, number of events per candidate prognostic factor, exploratory/confirmatory approach, follow-up length), participant characteristics (setting, inclusion and exclusion criteria, recruitment method, sociodemographic characteristics, details of treatment received, clinical tumor characteristics), candidate prognostic factors (number and type of prognostic factor, definition, method and timing of measurement of candidate prognostic factors, all unadjusted (simple) and adjusted (multivariable) associations reported between the prognostic factor and the outcome, with details on any adjustment factors that are used), outcomes (type of outcome, definition, measurement and timing of outcome assessment, time of outcome occurrence), and missing data (number per patient/per candidate prognostic factor, handling of missing data). The data extraction sheet will be pilot-tested to ensure its performance.

We will classify included studies according to the authors’ objectives, study design, definition of variables, and analysis (exploratory versus confirmatory intent). This information will be considered when interpreting the strength of the available evidence.

We will not consider individual patient data as the collection of such data is not feasible. Instead, we will contact the authors of the original publication in case of unclear or missing data.

If we identify several publications of the same study, we will consider the most recent and comprehensive one in the analysis.

All references will be managed and stored in EndNote X7.

### Assessment of risk of bias of included studies

Included studies will be critically appraised by two review authors independently, using the Quality in Prognosis Studies (QUIPS) tool, which is recommended by the Cochrane Prognosis Method Group for prognostic factor review questions. Discrepancies will be resolved through discussion and reaching a consensus. If necessary, a third author will be included. The QUIPS tool considers six domains of assessment: study participation, study attrition, prognostic factor measurement, outcome measurement, study confounding, and statistical analysis and reporting [20]. Results will be presented in a risk of bias table.

### Data analysis and synthesis

#### Measures of association

Hazard ratios (HRs) will be used to measure the relationship between the potential prognostic factor and tumor recurrence (time to event). We will extract all unadjusted and adjusted measures of association. An indirect estimation method will be used to calculate log HRs and their standard errors if they are not reported but adequate univariate analyses are available [21]. Odds ratios and relative risks will be used to estimate HRs as necessary [22]. For consistency, we will re-calculate associations to be in the same direction, as necessary, with associations above 1 indicating higher risk of relapse/worse prognosis. Standard errors will be calculated from confidence intervals and the individual study associations. Standard errors will be appropriately transformed to their natural logarithms if necessary [23].

#### Unit of analysis

Data retrieved from the primary studies is analyzed at the group level. We are aware that group or study level analyses can lead to biased assessments and have some limitations in revealing subset effects. But, in view of our broad approach including a set of factors investigated through a very long time span, we felt that an individual patient level analysis is not feasible for the present issue.

#### Dealing with missing data

We will include all studies investigating the association of clinical or pathological factors on tumor recurrence. Studies with missing data or not significant results will also be included. We will contact the corresponding author to request any important missing information or clarify questions, if necessary.

#### Publication bias

We will investigate publication bias by creating funnel plots for each meta-analysis containing at least ten studies. Publication bias will be assessed by visually examining the asymmetry of the funnel plot and by testing for asymmetry at the 10 % level, using Egger’s test for HRs and Peters’ test for odds ratios [24]. Furthermore, we will contact experts in the field to identify unpublished studies.

### Meta-analysis

Meta-analyses will only be conducted if valid data are available and data are sufficiently homogeneous for two or more studies. Data synthesis will be done separately with respect to differences in study design and study quality by excluding studies with low and/or unclear risk of bias.

In the meta-analyses, the random-effects generic inverse variance model will be used, which accounts for any between-study heterogeneity in the prognostic effect. Such heterogeneity is common in prognostic factor studies. Each meta-analysis will be summarized by the pooled estimate (the average prognostic factor effect), its 95 % confidence interval, the estimate of Tau^2^ (between-study variance), and a 95 % prediction interval for the prognostic effect in a single population [25, 26].

For continuous prognostic factors, meta-analyses will be conducted on the same scale. For dichotomous or categorical prognostic factors, we will group studies with similar cut points together to obtain meta-analysis results for each cut point as far as possible. To allow combination of as much data as possible, dichotomous associations will be computed from continuous and categorical measures of association from data in the study reports or provided by the study authors on request. We will explore the impact of data transformations using sensitivity analyses.

#### Assessment of heterogeneity

Forest plots as well as *I*^2^ and Tau^2^ will be used to assess and quantify statistical heterogeneity across the studies included in the meta-analyses (the estimate of between-study variance). The thresholds for interpretation of *I*^2^ will be in accordance with the definitions presented in the Cochrane Handbook for Systematic Reviews of Interventions [27]:

- 0 to 40 % might not be important
- 30 % to 60 % may represent moderate heterogeneity
- 50 % to 90 % may represent substantial heterogeneity
- 75 % to 100 % considerable heterogeneity

Clinical heterogeneity will be examined based on patient characteristics, clinical and pathological characteristics, and outcome measurement. Methodological heterogeneity will be examined based on study design and potential biases.

If it is not appropriate to combine the results in a meta-analysis (due to a small number of studies and/or if the interpretation of results would be difficult due to heterogeneity), they will be presented qualitatively by considering the strength and consistency of results. Therefore, the following schema will be used:

Strength of association will be defined based on effect size as weak (HR <1.5), moderate (HR 1.5–2.9), or strong (HR ≥3). Consistency of findings will be assessed using the following schema:

- Strong evidence of effect: Consistent findings (defined as >75 % of studies showing the same direction of effect) in multiple low risk of bias studies
- Moderate evidence of effect: Consistent findings in multiple high risk of bias and/or one study with low risk of bias
- Limited evidence of effect: One study available
- Conflicting evidence: Inconsistent findings across studies
- No evidence: No association between patient expectations and the outcome of interest

#### Sensitivity analyses

The robustness of results will be tested by sensitivity analyses.

Additional analyses will include the following comparisons:

- Exploratory versus confirmatory study results
- Retrospective versus prospective designs
- Categorized versus continuous data of the same factor
- Significant associations versus non-significant associations
- Adjusted versus non adjusted analyses
- Follow-up time (2 years, 3 years, 5 years, 10 years)

Data synthesis will be performed using Review Manager 5.3 provided by The Cochrane Collaboration [28]. Statistical methods not available in the Review Manager will be done using the statistical software R with the “meta” package [29, 30].

## Discussion

The value of prognostic markers for a tumor relapse in patients with clinically organ-confined stage I seminoma is discussed controversially in the medical literature. There is only little evidence from primary research, making decisions on adjuvant treatment for these patients difficult. Therefore, we will conduct a broad systematic review to identify prognostic factors that might be able to predict cancer recurrence in patients with clinical stage I seminoma. Current limitations of prognostic factor research is challenged by publication bias, reporting bias, poor statistical analyses, and inadequate reporting of methods and findings of primary studies. But within the increasing body of evidence, it becomes important to summarize the available information and thereby overcoming the limited validity of primary studies.

Systematic reviews of prognostic factors also provide several challenges, as they have been criticized for their limitation in providing relevant information due to missing quality appraisal of primary studies and poor adherence to standardized methodology and reporting. To overcome these challenges, we will follow the recommendations of conducting systematic reviews of prognostic studies, provided by the Cochrane Method Group of Prognosis Reviews and the PRISMA and REMARK reporting guidelines for systematic reviews and prognostic studies. Study appraisal for prognostic reviews is more difficult than for interventional reviews because there has been less research and funding in this field so far. We will use the QUIPS tool, recently described by Hayden et al. and recommended by the Cochrane Prognosis Methods Group [20].

Our results of the aggregation and critical appraisal of the available evidence will help to guide evidence-based treatment decision-making on patients with clinical stage I seminoma, allowing proper adjustment of therapies with regard to the individual patient’s risk. The discussion part of our study will address the limitations and strengths of this study. Our findings will be compared to results of other studies in this field. Our findings will furthermore help to formulate recommendations for future research, and clinical implications will be discussed.

## Appendix: search strategies

### Appendix 1

**Table 1:** OVID MEDLINE search strategy

1.	Testicular Neoplasms/ or Rete Testis/
2.	Germinoma/
3.	(testi* adj3 (cancer* or tumo* or neoplas* or carcinom* or malign*)).tw.
4.	“Neoplasms, Germ Cell and Embryonal”/
5.	(Germ* adj3 (cancer* or tumo* or neoplas* or carcinom* or malign*)).tw.
6.	Disease Progression/ or Risk Factors/ or Prognosis/
7.	Recurrence/ or Follow-Up Studies/ or Watchful Waiting/
8.	Neoplasm Recurrence, Local/
9.	Progression*.tw.
10.	Relaps*.tw.
11.	Recurrence*.tw.
12.	Seminom*.tw.
13.	Seminoma/
14.	1 or 2 or 3 or 4 or 5
15.	6 or 7 or 8 or 9 or 10 or 11
16.	12 or 13
17.	14 and 15 and 16

### Appendix 2

**Table 2:** Web of Science search strategy

# 7	#6 AND #1
# 6	#5 AND #4
# 5	TS = (Seminom*)
# 4	#3 OR #2
# 3	TS = (Germ* near (cancer* or tumo* or neoplas* or carcinom* or malign*))
# 2	TS = (testi* same (cancer* or tumo* or neoplas* or carcinom* or malign*))
# 1	TS = (Progression* OR Relaps* OR Recurrence*)

### Appendix 3

**Table 3:** CENTRAL search strategy

#1	(testi* near (cancer* or tumo* or neoplas* or carcinom* or malign*))
#2	(Germ* near (cancer* or tumo* or neoplas* or carcinom* or malign*))
#3	Seminom
#4	#1 or #2
#5	#4 and #3

## Abbreviations

ASCO: American Society of Clinical Oncology
AUA: American Urologic Association
CENTRAL: Cochrane Central Register of Controlled Trials
DGU: Deutsche Gesellschaft für Urologie
EAU: European Association of Urology
ESMO: European Society of Medical Oncology
HR: hazard ratio
OR: odds ratio
PRISMA: Preferred Reporting Items for Systematic Reviews and Meta-analyses
PROGRESS: Prognostic Research Strategy
QUIPS: Quality in Prognosis Studies
REMARK: Reporting Recommendations for Tumor Marker Prognostic Studies
